# Oncofertility as a Universal Right and a Global Oncology Priority

**DOI:** 10.1200/GO.19.00337

**Published:** 2020-03-02

**Authors:** Maria T. Bourlon, Antoinette Anazodo, Teresa K. Woodruff, Eva Segelov

**Affiliations:** ^1^ Instituto Nacional de Ciencias Médicas y Nutrición Salvador Zubirán, Mexico City, Mexico; ^2^ University of New South Wales, Sydney, Australia; ^3^ Feinberg School of Medicine, Northwestern University, Chicago, IL; ^4^ Monash Health and Monash University, Clayton, Victoria, Australia

In today’s era, where oncofertility is a firmly established discipline, why highlight this as a priority for the global oncology community? In the 4 years since the birth of *Journal of Global Oncology* (*JGO*), seven articles in this field have been published, reflecting the experience from 28 countries. These rate among the highest-cited papers.^1-7^

Oncofertility is an integral step in cancer management in high-income countries (HICs). With increasing cure and long-term survival rates of children, adolescent/young-adult men, and women of childbearing age, oncofertility is often included as a measure of quality practice.^8^

However, low- to middle-income countries (LMICs) continue to face many issues in the adoption and widespread implementation of oncofertility, from resources and access to expertise and cost. Of particular note are the dimensions of cultural and social effects of oncofertility. A major issue is inequity because of lack of publicly funded services, which restricts information available to patients and families regarding the range of options.^9^

The first article, “Creating a Global Community of Practice for Oncofertility,” published in 2015 in *JGO*, described the establishment of the Oncofertility Consortium in 2007.^1^ Funded by the National Institutes of Health, the Oncofertility Consortium originally aimed to join US centers that provided oncofertility care. In less than a decade, this group expanded to engage 43 countries from six different continents. Two years ago, to recognize the participation of 43 nations and to unify efforts around the globe, the Oncofertility Professional Engagement Network was formed. By connecting established networks in HICs with emerging efforts in LMICs, the acceptance of oncofertility on a global level has been advanced.

Important data were published in *JGO* by Oncofertility Professional Engagement Network members and collaborators, on the basis of two large surveys with responses from 40 fertility and cancer centers across 28 nations: 17 HICs and 11 LMICs. The “Survey of Fertility Preservation Options Available to Patients With Cancer Around the Globe” reported on access to oncofertility services.^2^ Barriers to implementation were recognized by 93% of respondents. The most common barrier reported was financial burden to patients (62%), followed by religious or cultural restrictions (61%), then by lack of specialized providers (24%). The “Survey of Third-Party Parenting Options Associated With Fertility Preservation Available to Patients With Cancer Around the Globe” reported significant and diverse educational and cultural attitudes.^3^ For example, in Tunisia, it was reported that unmarried women were fearful of losing their virginity after transvaginal procedures. Regarding adoption, the article reported that it is prohibited in Egypt, and in Chile it is denied for homosexual couples; in India, Iran, Turkey, Denmark, Portugal, the Netherlands, and Argentina, couples (heterosexual or homosexual) must have lived together for a prerequisite number of years to be considered. Of the 28 nations surveyed, egg donation is legal in 19; in 12 countries, it is accessible to heterosexual and homosexual married couples and in 17 countries, it is accessible to unmarried persons.

Despite these limitations, there is evidence that oncofertility practice is sought in LMICs. In 2018, Salama et al^6^ reported on the practices of nine countries with public health expenditure less than 4% of gross domestic product. Strikingly, a great variety of assisted reproductive techniques (including intrauterine insemination, in vitro fertilization, and intracytoplasmic sperm injection, as well as cryopreservation of sperm, embryos, and oocytes) were available in these countries. That said, approximately 90% of the services were provided in private centers and not covered by health insurance or public funding.

What can be done to improve the uptake of needed oncofertility services in LMICs? One concept is the development of oncofertility registries for LMICs that would allow comparison on a global scale. For HICs, the focus should be on documenting access for low socioeconomic and other disadvantaged groups. However, funding of sustainable registries poses a challenge. An innovative approach has been used by the Australasian Oncofertility Registry,^10^ an online prospective database collecting at the point of care on all pediatric and adult patients with cancer up to the age of 44 years, whether any oncofertility interventions occurred. The database commenced in 2014 in Australia and New Zealand, funded through a collaboration with Salesforce Pty Ltd, a US software company that oversees philanthropic cloud services as a platform for corporate social giving.^11^ It offers employees the opportunity to donate their information technology skills to support various causes.^10^ In real terms, this means that the Australasian Oncofertility Registry Web site is well maintained and can be easily modified without incurring large costs. This has facilitated expansion to India and the United States, with a view to reach further countries.

Global oncofertility will, of course, require culturally appropriate resources. A 2018 article, “Study of the Awareness of Adoption as a Family-Building Option Among Oncofertility Stakeholders in Japan,” highlighted the problems of using validated decision tools developed in Western countries.^4^ These include strategies such as oocyte retrieval, oocyte donation, use of a gestational carrier, and adoption. Yet donated ova and sperm are severely restricted in Japan, and adoption is rarely undertaken. Shiraishi et al^4^ demonstrated that culturally appropriate patient–provider education increased the use of adoption as an oncofertility preservation strategy in Japan.

Finally, the widely varied financial burden of oncofertility across LMICs, particularly for similar success rates, was presented by Salama et al in 2018, with one cycle of in vitro fertilization and intracytoplasmic sperm injection costing $USD 1,500 in India and $USD 10,000 in Chile.^7^ One could advocate on this basis for provision of high-tech procedures at low cost in LMICs, in much the same way as generic oncology drugs provide low-cost equivalent in countries such as India and across Africa.

Oncofertility is clearly a headline issue for patients, families, and communities in all countries. The development of resource-stratified oncofertility guidelines, along the lines of those produced by ASCO for treatment of various cancer subtypes,^12-15^ would be a welcome and timely initiative. More research is needed to define and optimize options on an equitable and sustainable basis, and produce cultural and context-specific patient and clinician information. *JGO* will proudly continue to publish academic papers from around the globe in this important field.
